# Medical Treatment of Obstructive Sleep Apnea in Children

**DOI:** 10.3390/jcm12155022

**Published:** 2023-07-30

**Authors:** Almala Pinar Ergenekon, Yasemin Gokdemir, Refika Ersu

**Affiliations:** 1 Division of Pediatric Pulmonology, Marmara University, 34890 Istanbul, Turkey; drpergenekon@hotmail.com (A.P.E.); yasemingokdemir@yahoo.com.tr (Y.G.); 2 Division of Respirology, Department of Pediatrics, Children’s Hospital of Eastern Ontario, University of Ottawa, Ottawa, ON K1N 6N5, Canada

**Keywords:** obstructive sleep apnea, persistent obstructive sleep apnea, children, medical treatment, PAP therapy, anti-inflammatory treatment, high flow nasal cannula

## Abstract

Obstructive sleep apnea (OSA) is characterized by recurrent complete or partial obstruction of the upper airway. The prevalence is 1–4% in children aged between 2 and 8 years and rising due to the increase in obesity rates in children. Although persistent OSA following adenotonsillectomy is usually associated with obesity and underlying complex disorders, it can also affect otherwise healthy children. Medical treatment strategies are frequently required when adenotonsillectomy is not indicated in children with OSA or if OSA is persistent following adenotonsillectomy. Positive airway pressure treatment is a very effective modality for persistent OSA in childhood; however, adherence rates are low. The aim of this review article is to summarize medical treatment options for OSA in children.

## 1. Introduction

Obstructive sleep apnea (OSA) is a syndrome involving upper airway dysfunction during sleep that is characterized by snoring and/or increased respiratory effort resulting from increased upper airway resistance and pharyngeal collapsibility [1,2,3]. It is estimated that 1–4% of children aged between 2 and 8 years have OSA. However, the prevalence may be as high as 80% in children with coexisting medical conditions, such as Trisomy 21 [4,5]. The prevalence of OSA is rising due to the increase in obesity rates in children [6].

Obstructive sleep apnea in children is caused by anatomic upper airway narrowing and/or increased upper airway collapsibility. Previous research showed that children with OSA had narrower pharyngeal airways when compared to control children during wakefulness, sedation, and paralysis. A smaller cranial base angle, longer lower facial height, mandibular retrognathia, a narrower dental arch, and various additional dental arch deformations, such as an anterior open bite, are cephalometric features that contribute to OSA. Children with OSA have dynamic inspiratory airway narrowing during tidal breathing in addition to the effect of increased soft tissue volumes on the static dimensions of the pharyngeal airway. The airway collapse can happen at different levels of the pharynx [7]. Drug-induced sleep endoscopy (DISE) refers to a flexible UA endoscopy performed during a sedative state that allows for a dynamic and three-dimensional evaluation of the entire upper airway. DISE helps to decide if there is a need for further surgical intervention other than adenotonsillectomy for treatment of OSA, specifically for post-adenontonsillectomy children, and what kind of intervention should be indicated for an individual patient, which may help to personalize treatment [8].

Depending on a child’s craniofacial morphology, tonsillar and adenoidal growth, and body habitus, as well as whether rhinitis symptoms are present, childhood OSA may consist of various overlapping phenotypes. Additionally, children with the same severity of OSA have variable end-organ morbidity. Compared to nonobese children, who typically present with impaired growth and adenotonsillar hypertrophy between the ages of 2 and 8 years, obese children may present later with symptoms that are more similar to those of adult OSA, such as excessive daytime sleepiness [9,10]. The OSA phenotype in children with complex diseases is determined by anatomical and functional abnormalities that are specific to each underlying disorder, such as Down syndrome and Prader Willi syndrome. It is essential that clinicians consider the symptoms, physical examinations, the presence of risk factors, and signs of end-organ morbidity to diagnose patients and develop a personalized management strategy [11,12,13].

Snoring, observed apneas, and gasping sounds while sleeping are signs of OSA [1]. Overnight polysomnography (PSG) is used to confirm the OSA diagnosis and the obstructive apnea hypopnea index (oAHI) is the main parameter used to diagnose and define the severity of OSA. However, PSG is associated with a significant financial and healthcare burden and it would be ideal to have a simple, reliable method for identifying children at high risk for OSA. For the purpose of pediatric OSA screening, several questionnaires have been developed. Determining a highly sensitive and focused questionnaire that is simple for patients/parents to complete and for clinicians to assess, however, is still a difficulty, and the sensitivity and specificity of these questionnaires are low [14]. However, pediatric sleep questionnaires are widely used not only to identify children at risk for OSA but also to evaluate response to treatment [15].

Polygraphies are simpler to perform and produce respiratory data comparable to a PSG. Overnight home or hospital use of respiratory polygraphies in children is more common now, much like adult sleep services. According to recent research, respiratory polygraphy can be successfully used with 81–87% of pediatric patients when it has been set up at a medical facility [16]. A recent European Respiratory Society technical standards paper summarized current data on the use of polygraphy in children for diagnosis of sleep-disordered breathing [17].

Although it has limitations, overnight oximetry can be a valuable tool for identifying children with OSA and determining the most urgent treatment needs if polygraphy or PSG are not available. These studies can be performed at home or in the hospital. The use of an appropriate oximeter is essential for correctly interpreting data. Averaging time is a key setting for evaluation of the oximeter’s diagnostic effectiveness. According to McGill’s system of evaluation, indicators of moderate to severe OSA include at least three clusters of desaturation events and at least three SpO_2_ drops below 90% in a nighttime oximetry recording [18]. Although this is not a very sensitive method of diagnosing OSA, it identifies children with moderate to severe disease successfully.

OSA is associated with cardiovascular morbidity and neurobehavioral impairments; therefore, it is important to diagnose and treat OSA in a timely manner [19,20,21]. Guidelines recommend treatment for children with an oAHI > 5 events/h and with an oAHI of 1–5 events/h in the presence of OSA morbidity or concomitant disease [22,23]. The objectives of OSA treatment in children are to reduce daytime symptoms, improve quality of life and sleep, and avoid short- and long-term consequences [24].

Even though the pathophysiology of pediatric OSA is heterogeneous, the overgrowth of the tonsils and adenoids, which restricts the upper airway during sleep, is the most frequent cause in children, even when associated obesity or complex disorders are present. Therefore, adenotonsillectomy (AT) is commonly the primary treatment option for children with OSA [1,25]. After surgery, OSA may relapse or persist in 21% to 73% of children [26,27]. Additional assessment and medical treatment strategies are frequently required when AT is not indicated or if there is persistent OSA after surgery, as well as when complex medical issues are present. The aim of this review is to summarize the current evidence for medical treatment of children with OSA (Table 1).

## 2. Anti-Inflammatory Treatment (Nasal Steroids/Montelukast/Oral Steroids) and Antibiotics

Many studies have investigated the effectiveness of anti-inflammatory medications, such as nasal steroids (NSs) or leukotriene receptor antagonists (montelukast), in children with mild to severe OSA since the pathophysiology of the condition has a significant inflammatory component [28]. Research has shown that pediatric adenotonsillar tissues contain glucocorticoid receptors, and children with OSA have increased levels of these receptors [29]. Additionally, leukotriene receptors were found to be expressed in adenotonsillar tissue surgically removed from children with OSA [30]. Therefore, it is plausible that anti-inflammatory medications may decrease the size of adenotonsillar tissue, leading to improvements in OSA.

The aim of NS use is to decrease the volume of adenoids via suppression of inflammation when adenotonsillectomy is contraindicated or in children with mild OSA [1]. A partial reduction in adenoidal hypertrophy has been observed with the administration of NSs for 4 to 6 weeks [31,32]. Sixty-two children underwent a double blind, randomized, controlled study comparing nasal budesonide with a placebo. Following a 6-week treatment with nasal budesonide, the oAHI decreased from 3.7 ± 0.3 to 1.3 ± 0.2 events/h in children with mild OSA. On the other hand, in the placebo group, the oAHI increased from 2.9 ± 0.4 to 4.0 ± 0.4 events/h (*p* <0.0001). Significant changes were seen in sleep macroarchitecture, such as sleep latency and the percentages of total sleep time spent in slow-wave sleep and rapid-eye-movement sleep (*p* < 0.05). Additionally, there was a long-lasting effect 8 weeks after the end of the treatment [32].

A recent double blind, randomized, controlled trial of NSs for the treatment of OSA in children included 134 children aged 5 to 12 years. Patients were randomized 2:1 to receive 3 months of NSs or a placebo. NS or placebo treatments for 9 months were then randomly reassigned to the children in the NS group. Changes in the oAHI at 3 months (median: −1.72 (interquartile range (IQR): −3.91 to 1.92) events/h) and 12 months (median: −1.2 (IQR: −4.22 to 1.71) events/h) were not different between the two groups (*p* = 0.7). OSA symptoms and neurobehavioral outcomes at 3 and 12 months were also similar between groups. Although there was a statistically significant decrease in the oAHI (7.2 (3.62 to 9.88) events/h to 3.7 (1.56 to 6.4) events/h, *p* = 0.39) in 38 children who received NSs for 12 months, this was not clinically significant [33]. Nasal irritation and bleeding are the two most common adverse effects of NSs. If NSs are administered for a prolonged period, there may be an increased risk of adrenal gland and growth suppression [34].

Gozal et al. included 64 children in a randomized, controlled study evaluating the effect of montelukast therapy. Of the 64 participants, 57 (89.0%) completed the 16-week trial with montelukast or a placebo, and among these, 42 were adherent to the assigned treatment (21 in the montelukast group and 21 in the placebo group). The study revealed that a 16-week treatment with montelukast significantly decreased the severity of OSA in children compared to the placebo. The AHI decreased from 9.2 ± 4.1 events/h to 4.2 ± 2.8 events/h (*p* < 0.0001) in the treatment group, whereas in the placebo group, the AHI increased from 8.2 ± 5.0 events/h to 8.7 ± 4.9 events/h. While 20 pediatric patients who received treatment (71.4%) experienced positive benefits, just 2 (6.9%) of the patients receiving the placebo showed decreases in the AHI (*p* <0.001). Similarly, the 3% Oxygen Desaturation Index (number of 3% reductions in SpO_2_ per hour of sleep) and arousal indices significantly improved in the treatment group, whereas no significant changes occurred in the placebo group [35].

According to a meta-analysis, five studies with 166 children that evaluated montelukast alone for pediatric OSA revealed a 55% improvement in the AHI (mean of 6.2 events/h pre-treatment vs. 2.8 events/h post-treatment), with improvement in the lowest oxygen saturation (LSAT) from 89.5% to 92.1%. Two studies with 502 children evaluating the effects of montelukast with NSs on pediatric OSA found a 70% improvement in the AHI (4.7 events/h pre-treatment vs. 1.4 events/h post-treatment), with an improvement in LSAT from 87.8% to 92.6% [36]. However, it should be noted that montelukast has significant adverse effects on mood and behavior, and the FDA has published a boxed warning for this medication [37]. Before initiation of treatment, clinicians should assess the risks and benefits and inform the families of these risks.

Since enlarged adenoids and tonsils are composed of hypertrophic lymphoid tissue, anti-inflammatory medications, such as systemic corticosteroids, have been evaluated for treatment of children with OSA. Evangelisti et al. evaluated the effect of systemic steroids in 28 children (mean age: 4.5 ± 1.8 years) with OSA. Fifteen children received oral betamethasone (0.1 mg/kg per day) in addition to NS therapy for 7 days in group one, while 13 children received NSs for 21 days in group two. The sleep clinical record score (12.6 ± 1.2 vs. 8.3 ± 1.1, *p* = 0.0001), oxygen desaturation index (11.7 vs. 3.0, *p* < 0.0001), oxygen desaturation time < 90% (1.75 vs. 0.0, *p* < 0.0001), oxygen desaturation events < 90% (25.5 vs. 1.0 *p* < 0.0001), and mean (95.3 ± 1.1 vs. 97.0 ± 0.8%, *p* = 0.0001) and minimum SpO2 (78.8 ± 6.3 vs. 89.2 ± 4.2, *p* = 0.001) improved in children treated with NSs and oral betamethasone. The authors recommended the use of systemic steroids as a bridging therapy prior to AT therapy in children with severe OSA [38].

Various microorganisms have been isolated from patients with chronic tonsillar hypertrophy. There has been limited research supporting the use of broad-spectrum antibiotics to treat pediatric OSA. In a study involving 22 children with OSA and adenotonsillar hypertrophy, researchers divided the patients into two groups. The first group, consisting of 11 children, received 30 days of azithromycin therapy, and the second group of 11 children received 30 days of placebo treatment. The AHI decreased by −0.97 ± 2.09 events/h in the azithromycin group but increased by 3.41 ± 3.01 events/h in the placebo group (*p* = 0.23). The baseline and maximum end-tidal carbon dioxide pressure, the baseline and LSAT, and the number of pathological central apneas did not change significantly. The authors concluded that broad-spectrum antibiotic therapy may not be an appropriate alternative to surgery and that larger studies are needed to determine the role of antibiotics in the treatment of OSA [39].

A paranasal sinus infection that lasts for more than three months is referred to as chronic sinusitis. Children with chronic sinusitis may have biofilms in their nasopharynx that serve as long-term reservoirs for bacteria that are resistant to common antibiotics. A previous study revealed that extensive biofilm formations on the adenoids of children with chronic sinusitis and those with obstructive sleep apnea raise the possibility that the generation of biofilms may be a virulence factor for the organisms that cause the disease [40]. Despite the fact that broad-spectrum oral antibiotics are frequently used to treat infections, chronic rhinosinusitis may not respond to antibiotic therapy with a permanent or sustained improvement. There is currently no agreement on the optimal treatment duration, organism coverage, or antibiotics due to the large range of aerobic and anaerobic organisms cultivated from the paranasal sinuses. However, high-dose antibiotics are typically used for a minimum of three weeks, whereas adenoidectomy can mechanically eliminate infection [40].

## 3. Positive Airway Pressure

It is important to consider that residual OSA may remain even after surgical intervention, especially in children with complex disorders, such as Trisomy 21, Prader–Willi syndrome, or obesity [27,41]. Children who are not candidates for surgery, most children with cranio-facial anomalies, and children with persistent OSA after adenotonsillectomy are usually started on positive airway pressure (PAP) therapy. A study by Kearney et al. found that the majority of adolescents with obesity (74%) had severe OSA (AHI ≥ 10 events/h) with a mean baseline AHI of 33.9 events/h. After AT, the AHI levels in the obese and control groups both showed clinically significant improvements with median changes of 18.3 events/h (*p* < 0.001) and 14.6 events/h (*p* < 0.001), respectively. A total of 48% of the obese adolescent patients had an AHI < 5 events/h on postoperative PSGs. However, compared to patients who were not obese, adolescents with obesity were seven times more likely to have moderate or severe persistent OSA (AHI > 5 events/h) after AT (*p* = 0.001). Adolescents with obesity had a considerably higher requirement for post-AT PAP therapy (37.1% of patients required PAP, *p*< 0.001) [42].

A device that can produce various levels of PAP, expressed in centimeters of water pressure, is used for PAP therapy. By using a pneumatic splint for the soft tissues of the upper airway, PAP treatment sustains airway stability throughout the breathing cycle [1]. It aims to regulate sleep architecture, increase sleep quality, and alleviate daytime symptoms caused by inadequate sleep.

A mask known as an interface connects the patient to the PAP treatment device. Finding an interface with a good fit that ensures comfort and optimal air leakage for the patient is crucial. However, there are no standards for choosing the right interface. Age and facial morphology are the most important factors when choosing an interface. This can be difficult, particularly for infants and children with asymmetry or facial deformity. Each interface has an intentional leak built in to avoid carbon dioxide rebreathing. An interface should have a good seal, minimal resistance to airflow, low dead space volume, and the optimal unintended leakage. Children can use nasal pillows and nasal and oronasal masks as interfaces for PAP therapy. Nasal masks are commonly preferred, as oronasal masks carry the risk of aspiration. Since nasal pillows fit right into the nares, they may be a good option for teens and are well tolerated. Children with OSA should be evaluated for nasal obstruction before initiating PAP therapy. If soft tissue obstruction is noted, medical treatment, such as NSs or montelukast, should be considered [35,36,43]. Improving nasal breathing can enhance the efficiency and tolerability of PAP therapy [44,45]. The nose and mouth are both covered by an oronasal interface. There are challenges when using an oronasal mask. The pressure from the mask on the jaw may produce posterior displacement and exacerbate the obstruction of the upper airway. Furthermore, if the child has muscle weakness or is young and unable to remove the mask in cases of vomiting, there is a danger of aspiration. Patients tend to tolerate the mask less well and are more likely to feel claustrophobic [46]. Furthermore, oronasal masks with the same pressure were not as effective as nasal masks in a study that evaluated adults with OSA using DISE [47]. However, there are limited data regarding the performance of interfaces in children [48]. In a study by Ramirez et al., no differences were detected in PAP adherence, correction of nocturnal gas exchange abnormalities, or leak values with the usage of nasal and oronasal masks in a retrospective analysis of 62 children (>2 years of age) [49].

The eyes, nose, and mouth are covered with a full-face mask. The pressure points for this contact are farther away than with normal interfaces, which is a benefit. Skin erythema and midface hypoplasia are mostly avoided. Given the significant dead space, patients should be clinically evaluated while using this mask to confirm that CO_2_ is not being re-breathed, especially in younger children. Additionally, if there is a risk of aspiration or if the child has increased oral secretions, full face masks should be avoided to prevent aspiration. These masks are mostly used in acute care of children with respiratory failure.

PAP therapy can be administered as continuous positive airway pressure (CPAP) or bi-level positive airway pressure (BPAP). CPAP prevents the collapse of the upper airway by continuously applying pressure at one level throughout the breathing cycle. It does not assist with the inspiration of the patient but improves gas exchange and oxygenation by increasing functional residual capacity [50]. BPAP provides assistance during inspiration by delivering cycling pressure. The ventilator’s high airflow rate augments the patient’s efforts to inhale. Therefore, it should be administered in accordance with the patient’s breathing efforts. The three main objectives of inspiratory positive airway pressure (IPAP) are to decrease the work of breathing, the respiratory rate, and PaCO_2_. The main objectives of the expiratory positive airway pressure (EPAP) are to increase oxygenation, decrease intrinsic PEEP, and remove upper airway obstruction. If the child needs a high expiratory pressure and cannot tolerate CPAP or has substantial hypoventilation that does not improve with CPAP use, BPAP can be used to treat OSA [51].

In BPAP devices, four different modes can be provided [46]. The mode of therapy is chosen depending on the patient’s breathing pattern and the underlying disorder. The spontaneous BPAP mode (BPAP-S) is used for children who are intolerant of CPAP at high pressures due to discomfort at exhalation. In this mode, each breath is started by the patient, higher inspiratory pressure and lower expiratory pressure are used, and there is no back-up rate. The spontaneous-timed BPAP mode (BPAP-ST) is used for children who present with mixed apnea, CPAP emergent central apnea, or persistent hypoventilation following resolution of OSA with CPAP. In this mode, a back-up rate, which is usually 2–4 breaths lower than the patient’s own respiratory rate, is employed [52]. The patient initiates the breaths and the device only delivers a breath when the patient’s spontaneous respiratory rate drops below the pre-set back-up rate. In the pressure control mode, there is a set inspiratory time for both ventilator and spontaneous breaths. In the timed mode, which is rarely used in children, the device controls the patient’s breathing rate and inspiratory times regardless of the patient effort. PAP treatment has been linked to significant clinical advantages, such as decreased risk of cardiovascular disease and reduction in insulin resistance [53,54,55].

A novel mode of ventilation known as the volume-assured pressure support (VAPS) BPAP mode provides automatically titrating pressure support that is designed to achieve a tidal volume goal. It has been shown to be useful for children with obesity hypoventilation syndrome and congenital central hypoventilation syndrome [52].

A titration sleep study with PAP titration usually precedes the beginning of PAP therapy. In order to prevent respiratory events and improve gas exchange, pressure/mode modifications are implemented during the sleep study. The goals for PAP titration include <2 obstructive apneas per hour, ETCO_2_ < 55 mmHg and not greater than 50 mmHg for >10 min, SpO_2_ ≥ 94%, and minimal paradoxical breathing and flow limitation [51]. Additionally, there are auto-adjusting CPAP/BPAP devices that utilize a unique algorithm. These devices might be useful when the severity of OSA is dependent on body posture and/or sleep stage [56]. CPAP titration with an auto-CPAP device in the home environment can be considered for children when access to a sleep laboratory is limited [57].

After initiation of PAP therapy, regular follow-up is necessary to ensure adequate therapy as the efficacy of PAP therapy is limited by low adherence. In children, physical discomfort and/or fear of the device may cause low adherence. Usage in the first week of treatment may predict longer-term use, and monitoring adherence in the first week of treatment and intervening in cases of low adherence may improve long-term CPAP use [58]. The largest PAP adherence analysis of pediatric patients with OSA was published in 2020 [59]. A total of 20,553 patients with a mean age of 13 years met the eligibility criteria and had accessible data. Based on 90 days of monitoring data, 12,699 patients (61%) used PAP continuously. However, only 46.3% of the cohort met the Centers for Medicare and Medicaid Services’ adherence requirements after 90 days. This adherence was poorer than that shown in the results from studies using similar methodologies to measure adherence in adults. Additionally, this study suggested that children between the ages of 4 and 6 years and adolescents between the ages of 15 and 18 years might require more assistance than other age groups, necessitating age-specific behavioral interventions. As children spend more hours in sleep, using adult criteria for adherence may not be sufficient to prevent adverse consequences in children with OSA.

Developmental delay, female gender, and younger age are associated with better PAP compliance [60,61]. A recent study compared the efficacy and challenges of PAP therapy adherence in infants and school-aged children with OSA [62]. A total of 41 infants and 109 school-aged children were included in the study. After PAP titration, infants’ oAHI levels decreased on average by 92.1%, while school-aged children’s oAHI levels decreased on average by 93.4% (0 = 0.67). The same types of challenges for adherence were reported in infants and school-aged children, with behavioral issues being the most prevalent in both populations. Another study included 137 typically developing (TD) children and 103 children with developmental disabilities (DDs). At 3 and 6 months, the percentage of nights when devices were used was significantly higher for children with DDs (*p* = 0.01, *p* = 0.003, respectively). Hours of usage on nights when the devices were used at three and six months were similar between groups (DD group = 5.0, TD group = 4.6, *p* = 0.71; DD group = 6.4, TD group = 5.7, *p* = 0.34, respectively). Higher PAP was strongly predictive for hours of usage in both groups at 6 months, while higher median neighborhood income and titration at or before 6 months were significantly predictive of percentage of nights when devices were used [63].

In addition to the assistance provided by medical staff, the patient’s environment and family at home are crucial for adherence. Marcus et al. found a high drop-out rate (35%), consistently low overnight use duration (5.3 h/night), and significant over-reporting of compliance by families in research on PAP adherence in children [64]. Another study on PAP compliance among pediatric patients revealed that <60% of patients adhered to recommended schedules ranging from 4.0 to 5.2 h per night [60]. Facemask discomfort contributed to low compliance. Teenagers’ adherence to PAP therapy can be encouraged by support groups, phone applications, behavioral therapy, and motivational interviewing methods [65]. A systematic review showed that children with caregiver support had significantly longer CPAP use per night (by 86.60 min) and significantly higher percentages of CPAP usage for more than 4 h/night (by 18.10%) than those without caregiver support. Although data showing better compliance with BPAP mostly come from studies on adults, there is some evidence that supports this in children as well. When compared to those who received CPAP therapy, children who received BPAP therapy had an 18.17 times higher likelihood of having good PAP adherence [66]. Adherence to CPAP or BPAP therapy should be monitored by using the device software [67].

Although adverse effects of PAP therapy are mostly minor, it is important to address these issues to improve adherence. Air leaks frequently cause discomfort. Abdominal distension, oronasal dryness, eye irritation, and pressure sores on the nasal bridge caused by the masks may be seen in children. It is often recommended to use a humidifier to reduce discomfort caused by cold, dry air. Additionally, the midface may flatten as a result of the mask’s continuous pressure on the growing facial tissues [68]. It is very important to make sure that the mask fits gently on the face rather than being firmly fixed to reduce the impact on the midface.

## 4. High-Flow Nasal Cannula Therapy

High-flow nasal cannula (HFNC) treatment has been used to treat neonates with respiratory distress linked to prematurity in neonatal intensive care units with varying but generally positive effects, including decreased effort in breathing and lower rates of respiratory failure [69,70]. HFNC therapy delivers humidified and heated air at a high flow rate via nasal prongs. Continuous positive pressure is produced in the airways by the HFNC, and oxygen is continuously pushed into the upper airways at a rate that is higher than the typical inspiratory flow rate (approximately 4–7 cm H_2_O at maximal flow rates), preventing upper airway collapse [71,72,73].

Children with OSA who cannot tolerate the CPAP masks may benefit from HFNC treatment [74]. Pediatric OSA has been successfully treated with high-flow heated, humidified nasal air [28,75,76,77]. Ten children (1–18 years) with obstructive sleep apnea determined to be CPAP-intolerant by their caregivers were included in a study by Hawkins et al. High-flow humidified room air was initially delivered at rates of 5 to 15 L/min with pediatric or adult-sized cannulas and then gradually increased. If hypoxemia or desaturations persisted at the maximum rate of room air, oxygen was added. This study showed that HFNCs can successfully treat moderate to severe obstructive sleep apnea in CPAP-intolerant children (the oAHI improved from 11.1 to 2.1 events/h, *p* = 0.002; the obstructive hypopnea index improved from 9.9 to 0.5 events/h, *p* = 0.002) [78].

A total of 22 patients (mean age: 12.8 months) who had persistent OSA after adenotonsillectomy with CPAP intolerance, whose caregivers refused to use CPAP, or who were not good surgical candidates were included in a retrospective study. The HFNC titration study was performed an average of 128 days after the diagnostic sleep study. The oAHI decreased from 28.9 to 2.6 events/h, the oAI decreased from 14.4 to 0.4 events/h, and the OHI decreased from 14.5 to 2.2 events/h. In this study, HFNCs not only improved sleep parameters but were also well tolerated. The majority of patients adhered to their HFNC therapy throughout a 12-month period of home use. Cannula dislodgement was the most common complication of home HFNC therapy, as observed in 12 patients (63%). The authors suggested that HFNCs could be used as a temporary bridge therapy to treat OSA before surgery or an alternative long-term treatment [79]. Although HFNC use at home is currently limited and costly, use of HFNCs for OSA at home may become an option in the near future.

## 5. Positional Therapy

In positional OSA (POSA), the OSA occurs mostly while sleeping in the supine position, and this is known to affect 19–58% of children with OSA [80,81]. Obese children may have more profound upper airway obstruction during supine sleep, as greater fat deposition in the pharyngeal region results in a smaller upper airway [82]. POSA occurs when the supine AHI is at least two times higher than the non-supine AHI [80]. Decreased craniofacial volume, decreased lung volume, and the inability of the airway dilator muscles to prevent airway collapse during an occlusion may occur in the supine position [83,84,85]. A study by Selvadurai et al. evaluated 112 obese children with PSG, and 43 (38%) children had OSA. Among those with OSA, 25 (58%) had POSA (mean age: 14.6 ± 2.3 years; mean body mass index: 37.7 ± 7.6 kg/m2; 68% male) and 18 (42%) had non-POSA (mean age: 13.9 ± 2.8 years; mean body mass index: 37.9 ± 7.2 kg/m2; 78% male). Among those with POSA, 13 (52%) had mild OSA, 7 (28%) had moderate OSA, and 5 (20%) had severe OSA. There were no significant differences in age, sex, or anthropometric measures between the POSA and non-POSA groups. However, older children were more likely to have POSA; 88% of the children with POSA were 12 years or older (*p* = 0.41) [81].

The capacity to sustain comfortable non-supine sleep is a requirement for positional therapy (PT) in children. A belt worn around the chest with pillows on the back to stop children from adopting the supine posture may be an effective treatment option for POSA. For children with persistent OSA, PT can be a simple, cheap, and low-risk treatment option. Children between the ages of 4 and 18 years with POSA who had a baseline PSG and a second PSG to assess the effectiveness of a positional device were included in a study by Xiao et al. [83]. The median body mass index z-score was 1.6. Compared to the baseline data, PSG results obtained while using a positional device showed reductions in the median oAHI (15.2 vs. 6.7 events/h, respectively; *p* = 0.004) and in the percentage of total sleep time in supine position (54.4 vs. 4.2 h, respectively; *p* = 0.04) [86]. More studies are needed but, considering the cost effectiveness and non-invasive nature of this treatment, positional therapy may be a viable option for children with POSA.

## 6. Myofunctional Therapy

Persistent oral breathing during sleep may affect the strength of the tongue and orofacial muscles, leading to abnormal airway development and OSA [39]. Myofunctional therapy (MT) is based on isotonic and isometric exercises that enhance the orofacial tissues’ coordination and strength [87]. MT involves multiple tongue, soft palate, and facial muscle exercises. Daily practice of these exercises strengthens the orofacial muscles. A study by Villa et al. revealed that MT improved tongue tone and decreased respiratory symptoms and oral breathing during sleep in all 36 children with sleep-disorder breathing [88]. Another investigation on children with mild persistent OSA revealed that MT decreased OSA severity in 14 children after 2 months compared to 13 controls (decrease in oAHI of 58% in the MT group vs. 6.9% in the control group; *p* = 0.004). However, more studies are needed with children before MT can be widely used.

## 7. Dental Procedures

Although it has been suggested that a subset of craniofacial characteristics, including increased facial height, retrognathia, and a higher mandibular angle, may be more frequently present in children with OSA, a recent meta-analysis of nine studies revealed that, although a certain subgroup of pediatric OSA patients showed higher rates of specific craniofacial characteristics, this was not consistent across studies [89]. The authors concluded that there is insufficient evidence to report a link between pediatric cases of OSA and craniofacial morphology [90].

Rapid maxillary expansion (RME) is a type of orthodontic treatment that widens the hard palate by expanding the airway using a dental device, beginning around age four and continuing until the midpalatal suture fuses in adolescence [8]. According to the results of a meta-analysis, improvements in AHI and lowest oxygen saturation levels were observed in children who underwent RME treatment, particularly at short-term (3-year) follow-up [91]. Thirty children with OSA were included in a study by Hoxha et al.; fifteen were enrolled as the control group, while fifteen received semi-rapid maxillary expansion (SRME) orthodontic treatment for 5 months. In addition to respiratory parameters, the pharyngeal area, dental arch, postero-anterior widths, and OSA biomarker levels (ORM2, FABP4, perlecan, gelsolin, KLK1, and uric acid) in serum and urine were measured. The AHI decreased from 2.5 to 1.79 events/h (28% decrease, *p* < 0.05) after a 5-month treatment period, while it decreased from 2.67 to 1.8 events/h (33% decrease, *p* < 0.05) in the control group [92]. A recent systematic review compared the effect of RME to watchful waiting or alternative therapies for pediatric OSA and included five trials. Only one randomized clinical trial compared RME with watchful waiting. The other four studies (three of them were non-randomized) compared RME with the gold-standard therapy AT. There was no evidence that RME treatment significantly outperformed watchful waiting in patients with pediatric OSA in this systematic review. It was concluded that the non-homogeneous distribution of confounders and inadequate designs made comparisons between treatment alternatives difficult. Further studies are needed to compare the effect of RME to that of watchful waiting [93].

## 8. Weight Loss

Obesity is a risk factor for developing OSA [94]. OSA has been diagnosed in 13–59% of obese children [95]. A total of 139 children with a median age of 4.5 years were included in a study where 25 of the children were overweight and 21 were obese. The study revealed that, regardless of age or prior upper airway surgery, a one-unit increase in BMI z-score was associated with 67% increased odds of circumferential collapse during drug-induced sleep endoscopy (DISE). The authors reported that this circumferential pattern may be less sensitive to AT and that nonsurgical treatments, such as CPAP and weight loss, may be necessary in these patients. Other treatment approaches should be started until enough weight loss has been achieved since this treatment modality requires a motivated patient and family and the process might be slow [1]. The success rates and cure rates of DISE-directed treatment were similar in children who were normal weight, overweight, and obese [96].

In a study of 339 obese children with a median age of 15.4 years, after an average 32% decrease in BMI z-score, 80% of the children showed improved sleep-disordered breathing [97]. Ten studies conducted on participants with an age range of 10–19 years were included in a meta-analysis that evaluated the prevalence and severity of OSA in obese children, as well as the impact of weight loss strategies. There was an improvement in OSA prevalence post-intervention, and OSA was cured in 46.2–79.7% of the participants. The meta-analysis showed significant reductions in the AHI (effect size: −0.51, 95%CI −0.94 to −0.08, *p* = 0.019) and oxygen desaturation index (effect size: −0.28, 95%CI = −0.50 to −0.05, *p* = 0.016). Seventy-five percent of the studies reported improved sleep duration in participants with OSA [98].

As management of childhood obesity with diet and exercise alone is challenging, several drugs (metformin, glucagon-like peptide-1 receptor agonists, and phentermine-topiramate) have been studied to treat pediatric obesity. Liraglutide and exenatide are the most commonly investigated medications in terms of weight loss in adults. A randomized, double-blind, placebo-controlled trial evaluated the efficacy and safety of subcutaneous liraglutide 3.0 mg as an addition to lifestyle therapy for weight management in adolescents with obesity. Individuals (age 12 to 18 years) with obesity and a poor response to lifestyle changes alone were included to the study. There were 126 participants in the placebo group and 125 in the liraglutide group. With an estimated difference of 0.22, liraglutide exceeded the placebo in terms of the BMI standard deviation score change from baseline at week 56. A decrease in BMI of at least 5% was seen in 43.3% of the liraglutide group and 18.5% of placebo group participants; a decrease in BMI of at least 10% was seen in 33% and 9% of participants, respectively [99].

The phentermine/topiramate extended-release capsule is a fixed-dose combination of phentermine and topiramate developed for the treatment of obesity, sleep apnea syndrome, and type 2 diabetes mellitus. The once-daily formulation of phentermine and topiramate is designed to combat obesity by decreasing appetite and increasing satiety. Phentermine/topiramate has received its first US approval for chronic weight management in pediatric patients aged ≥12 years with a BMI in the 95th percentile or greater for age and sex in combination with a low-calorie diet and increased physical activity. Clinical development of phentermine/topiramate for sleep apnea syndrome and type 2 diabetes in obese patients is ongoing in the US, and it may be a treatment option in children with OSA related to obesity in the future [100].

Bariatric surgery has been found to be beneficial in decreasing excess weight and alleviating comorbidities in adolescents with severe obesity. A retrospective study was conducted in adolescents with morbid obesity who underwent laparoscopic adjustable gastric band (LAGB) surgery between 1995 and 2018. Fifty-nine adolescents (mean age: 17.7 ± 1.5 years, mean BMI: 40.9 ± 6.4) were included in the study. Sixty-nine percent of the adolescents with morbid obesity who had OSA at baseline showed resolution of the OSA at one-year follow up after bariatric surgery (the mean BMI was lower at 34.4 ± 6.3 kg/m^2^). In seven adolescents with OSA (mean age: 17.8 years), bariatric surgery reduced the oAHI from 13 ± 6.9 events/h to 4.5 ± 2.5 events/h (*p* < 0.05) at 3 weeks post-operatively [101]. However, it should be noted that there are limited data on the long-term efficacy and safety of bariatric surgery in adolescents.

Considering the significant effects of obesity on OSA and the poor response to adenotonsillectomy in children with obesity, weight loss should be part of the treatment plan for all children with obesity and OSA.

## 9. Hypoglossal Nerve Stimulation

The recurrent collapse of the upper airway during sleep is a hallmark of obstructive sleep apnea. The contraction of the upper airway dilator muscles maintains the patency of the upper airways. Although there are numerous muscles that dilate the upper airways, the most significant upper airway dilator muscle is the genioglossus muscle. The hypoglossal nerve innervates the genioglossus muscle, and hypoglossal nerve stimulation has been successfully used with adults with moderate to severe OSA who cannot tolerate CPAP therapy [102].

OSA is common in people with Down syndrome, with a prevalence of 55–97% and a high risk of persistent OSA following adenotonsillectomy, and this population usually have low adherence rates to CPAP therapy [103]. Therefore, hypoglossal nerve stimulation use may be helpful in children with Down syndrome and persistent OSA who have low adherence to CPAP therapy. In a meta-analysis of nine articles involving 106 adolescents with Down syndrome and OSA, there was an improvement in the AHI by at least 50% when patients were treated with hypoglossal nerve stimulation. Participants also showed improvements in the OSA-18 (a validated, disease-specific quality of life instrument for OSA) and in daytime sleepiness measured with Epworth Sleepiness Scale questionnaires [104].

A recent study investigated four participants who underwent hypoglossal nerve implantation by age 13 and completed at least 44 months of follow-up. Over the follow-up period, all four participants’ AHI levels remained at least 50% lower than they were at baseline. Two participants had persistent, moderate OSA despite stimulation therapy. The other two participants achieved 100% reductions in AHI levels with stimulation therapy; when they underwent split-night sleep studies, the severe OSA persisted with the device turned off [105,106].

## 10. Novel Pharmacotherapeutics

A selective norepinephrine reuptake inhibitor, atomoxetine, has been used to treat both adults and children with attention deficit hyperactivity disorder. Raising the norepinephrine content in the brainstem during sleep could activate the upper airway motorneurons to levels equivalent to those reported during wakefulness. An in vitro experiment revealed that atomoxetine also blocks G-coupled inwardly rectifying potassium channels, which are important in pharyngeal hypotonia during sleep. Oxybutynin, an antimuscarinic with strong affinity for all muscarinic receptors, is used to treat overactive bladder. Acetylcholine affects the hypoglossal motor nucleus in a variety of ways, with muscarinic-mediated genioglossus suppression typically outweighing nicotinic stimulation. Muscarinic blockade may increase the concentration of acetylcholine for nicotinic receptors and decrease the inhibitory effect of acetylcholine on upper airway muscle tone during REM sleep, collaborating with norepinephrine in the stimulation of upper airway dilator muscles. The hypoglossal motor nucleus expresses the inhibitory muscarinic receptor that oxybutynin antagonizes. This receptor is crucial for controlling the activity of the hypoglossal nerve [107,108]. Twenty adults (median age of 53 (46–58) years and BMI of 34.8 (30.0–40.2) kg/m^2^) participated in a randomized, placebo-controlled, double-blind crossover trial that compared 80 mg of atomoxetine and 5 mg of oxybutynin (ato-oxy) given before sleep versus a placebo for one night. This combination therapy lowered the AHI by 63% (34–86%) from 28.5 (10.9–51.6) events/h to 7.5 (2.4–18.6) events/h (*p* = 0.001) and increased genioglossus muscle responsiveness [107]. A trial is currently being conducted to investigate the effectiveness and safety of treating persistent OSA in children with Down syndrome with atomoxetine and oxybutynin (NCT04115878).

In conclusion, although adenotonsillectomy remains the primary treatment for children with OSA, there are medical treatment options that can be considered. As we acquire increased understanding of the phenotypes and endotypes of OSA in children, it will be possible to use the existing and emerging therapies in an individualized fashion.

## Figures and Tables

**Table 1:** Medical treatment modalities for obstructive sleep apnea in children.

	Indications and Benefits	Challenges
Anti-inflammatory treatment Nasal steroids/oral montelukast/oral steroids	Children with symptoms of allergic or non-allergic rhinitis may benefit. Anti-inflammatory medications may decrease the size of adenotonsillar tissue, leading to improvement in OSA.	Follow up is necessary Efficacy has to be evaluated due to variety in responses Nasal irritation and bleeding may occur with nasal steroids Montelukast can have adverse effects on behavior and mood
Antibiotics	Currently not recommended	Larger studies are needed to determine the role of antibiotics in the treatment of OSA
Positive airway pressure therapy	Children with moderate to severe OSA who are not candidates for surgery and children with persistent OSA after surgical intervention may benefit	Requires all-night and long-term use Low adherence rates It may be associated with midface hypoplasia
High-flow nasal cannula therapy	Children who cannot tolerate CPAP may benefit from HFNC treatment	Rarely covered by health insurance in many countries No home units recording adherence and no proper alarms
Positional therapy	Can be considered in children with persistent OSA Can be a simple, cheap, and low-risk treatment option	Requires all-night and chronic use Larger studies are needed to demonstrate its effectiveness No adherence data
Myofunctional therapy	Currently not routinely recommended	Requires training and daily exercises
Dental procedures	Recommended in children with narrow transversal maxillary arch, who collaborate in the expansion by turning screws to widen the airway and improve OSA	Effects not clear Long treatment duration Mostly not covered by insurance companies
Weight loss	Efficacious in treating OSA associated with obesity in children If medical treatment of obesity is not achieved, surgical options can be considered	Other treatment modalities should be initiated until enough weight loss has been achieved

## Data Availability

Data available in a publicly accessible repository.

## Abbreviations

OSA	obstructive sleep apnea
AHI	apnea–hypopnea index
oAHI	obstructive apnea hypopnea index
AT	adenotonsillectomy
NCS	nasal corticosteroid
LSAT	lowest oxygen saturation
PAP	positive airway pressure
CPAP	continuous positive airway pressure
BPAP	bi-level positive airway pressure
BPAP-ST	spontaneous-timed BPAP mode
BPAP-S	spontaneous BPAP mode
IPAP	inspiratory positive airway pressure
EPAP	expiratory positive airway pressure
VAPS	volume-assured pressure support
HFNC	high-flow nasal cannula
POSA	positional OSA
PT	positional therapy
MT	myofunctional therapy
RME	rapid maxillary expansion
SRME	semi-rapid maxillary expansion
BMI	body mass index

## References

[1] Marcus C.L., Brooks L.J., Draper K.A., Gozal D., Halbower A.C., Jones J., Schechter M.S., Ward S.D., Sheldon S.H., Shiffman R.N. (2012). Diagnosis and management of childhood obstructive sleep apnea syndrome. Pediatrics.

[2] Javaheri S., Barbe F., Campos-Rodriguez F., Dempsey J.A., Khayat R., Javaheri S., Malhotra A., Martinez-Garcia M.A., Mehra R., Pack A.I. (2017). Sleep Apnea: Types, Mechanisms, and Clinical Cardiovascular Consequences. J. Am. Coll. Cardiol..

[3] Kaditis A.G., Alvarez M.L.A., Boudewyns A., Alexopoulos E.I., Ersu R., Joosten K., Larramona H., Miano S., Narang I., Trang H. (2015). Obstructive sleep disordered breathing in 2- to 18-year-old children: Diagnosis and management. Eur. Respir. J..

[4] Lumeng J.C., Chervin R.D. (2008). Epidemiology of Pediatric Obstructive Sleep Apnea. Proc. Am. Thorac. Soc..

[5] Lee C.-H., Hsueh W.-Y., Lin M.-T., Kang K.-T. (2018). Prevalence of Obstructive Sleep Apnea in Children With Down Syndrome: A Meta-Analysis. J. Clin. Sleep Med..

[6] Arens R., Muzumdar H., Wootton D.M., Sin S., Luo H., Yazdani A., McDonough J.M., Wagshul M.E., Isasi C.R. (2010). Childhood obesity and obstructive sleep apnea syndrome. J. Appl. Physiol..

[7] Vos W.G., De Backer W.A., Verhulst S.L. (2010). Correlation between the severity of sleep apnea and upper airway morphology in pediatric and adult patients. Curr. Opin. Allergy Clin. Immunol..

[8] Ersu R., Chen M.L., Ehsan Z., Ishman S.L., Redline S., Narang I. (2022). Persistent obstructive sleep apnoea in children: Treatment options and management considerations. Lancet Respir. Med..

[9] Gozal D., Kheirandish-Gozal L. (2012). Childhood obesity and sleep: Relatives, partners, or both a critical perspective on the evidence. Ann. N. Y. Acad. Sci..

[10] Au C.T., Zhang J., Cheung J.Y.F., Chan K.C.C., Wing Y.K., Li A.M. (2019). Familial aggregation and heritability of obstructive sleep apnea using children probands. J. Clin. Sleep Med..

[11] Kamal M., Tamana S.K., Smithson L., Ding L., Lau A., Chikuma J., Mariasine J., Lefebvre D.L., Subbarao P., Becker A.B. (2018). Phenotypes of sleep-disordered breathing symptoms to two years of age based on age of onset and duration of symptoms. Sleep Med..

[12] Tan H., Kaditis A.G. (2021). Phenotypic variance in pediatric obstructive sleep apnea. Pediatr. Pulmonol..

[13] Gaines J., Vgontzas A.N., Fernandez-Mendoza J., Bixler E.O. (2018). Obstructive sleep apnea and the metabolic syndrome: The road to clinically-meaningful phenotyping, improved prognosis, and personalized treatment. Sleep Med. Rev..

[14] Abumuamar A.M., Chung S.A., Kadmon G., Shapiro C.M. (2017). A comparison of two screening tools for paediatric obstructive sleep apnea. J. Sleep Res..

[15] Pabary R., Goubau C., Russo K., Laverty A., Abel F., Samuels M. (2018). Screening for sleep-disordered breathing with Pediatric Sleep Questionnaire in children with underlying conditions. J. Sleep Res..

[16] Ioan I., Weick D., Schweitzer C., Guyon A., Coutier L., Franco P. (2020). Feasibility of parent-attended ambulatory polysomnography in children with suspected obstructive sleep apnea. J. Clin. Sleep Med..

[17] Riha R.L., Celmina M., Cooper B., Hamutcu-Ersu R., Kaditis A., Morley A., Pataka A., Penzel T., Roberti L., Ruehland W. (2023). ERS technical standards for using type III devices (limited channel studies) in the diagnosis of sleep disordered breathing in adults and children. Eur. Respir. J..

[18] Trucco F., Rosenthal M., Bush A., Tan H.-L. (2019). The McGill score as a screening test for obstructive sleep disordered breathing in children with co-morbidities. Sleep Med..

[19] Hunter S.J., Gozal D., Smith D.L., Philby M.F., Kaylegian J., Kheirandish-Gozal L. (2016). Effect of Sleep-disordered Breathing Severity on Cognitive Performance Measures in a Large Community Cohort of Young School-aged Children. Am. J. Respir. Crit. Care Med..

[20] Narang I., McCrindle B.W., Manlhiot C., Lu Z., Al-Saleh S., Birken C.S., Hamilton J. (2018). Intermittent nocturnal hypoxia and metabolic risk in obese adolescents with obstructive sleep apnea. Sleep Breath..

[21] Baker-Smith C.M., Isaiah A., Melendres M.C., Mahgerefteh J., Lasso-Pirot A., Mayo S., Gooding H., Zachariah J. (2021). Sleep-Disordered Breathing and Cardiovascular Disease in Children and Adolescents: A Scientific Statement From the American Heart Association. J. Am. Heart Assoc..

[22] Accardo J.A., Shults J., Leonard M.B., Traylor J., Marcus C.L. (2010). Differences in Overnight Polysomnography Scores Using the Adult and Pediatric Criteria for Respiratory Events in Adolescents. Sleep.

[23] Berry R.B., Budhiraja R., Gottlieb D.J., Gozal D., Iber C., Kapur V.K., Marcus C.L., Mehra R., Parthasarathy S., Quan S.F. (2012). Rules for Scoring Respiratory Events in Sleep: Update of the 2007 AASM Manual for the Scoring of Sleep and Associated Events. J. Clin. Sleep Med..

[24] Wang J.J., Imamura T., Lee J., Wright M., Goldman R.D. (2021). Continuous positive airway pressure for obstructive sleep apnea in children. Can. Fam. Physician.

[25] Mitchell R.B., Archer S.M., Ishman S.L., Rosenfeld R.M., Coles S., Finestone S.A., Friedman N.R., Giordano T., Hildrew D.M., Kim T.W. (2019). Clinical Practice Guideline: Tonsillectomy in Children (Update). Otolaryngol. Head Neck Surg..

[26] Redline S., Amin R., Beebe D., Chervin R.D., Garetz S.L., Giordani B., Marcus C.L., Moore R.H., Rosen C.L., Arens R. (2011). The Childhood Adenotonsillectomy Trial (CHAT): Rationale, Design, and Challenges of a Randomized Controlled Trial Evaluating a Standard Surgical Procedure in a Pediatric Population. Sleep.

[27] Bhattacharjee R., Kheirandish-Gozal L., Spruyt K., Mitchell R.B., Promchiarak J., Simakajornboon N., Kaditis A.G., Splaingard D., Splaingard M., Brooks L.J. (2010). Adenotonsillectomy outcomes in treatment of obstructive sleep apnea in children: A multicenter retrospective study. Am. J. Respir. Crit. Care Med..

[28] Gozal D., Tan H.-L., Kheirandish-Gozal L. (2013). Obstructive sleep apnea in children: A critical update. Nat. Sci. Sleep.

[29] Goldbart A.D., Tal A. (2008). Inflammation and sleep disordered breathing in children: A state-of-the-art review. Pediatr Pulmonol..

[30] Dayyat E., Serpero L.D., Kheirandish-Gozal L., Goldman J.L., Snow A., Bhattacharjee R., Gozal D. (2009). Leukotriene Pathways and In Vitro Adenotonsillar Cell Proliferation in Children With Obstructive Sleep Apnea. Chest.

[31] Berlucchi M., Salsi D., Valetti L., Parrinello G., Nicolai P. (2007). The Role of Mometasone Furoate Aqueous Nasal Spray in the Treatment of Adenoidal Hypertrophy in the Pediatric Age Group: Preliminary Results of a Prospective, Randomized Study. Pediatrics.

[32] Kheirandish-Gozal L., Gozal D. (2008). Intranasal Budesonide Treatment for Children With Mild Obstructive Sleep Apnea Syndrome. Pediatrics.

[33] Tapia I.E., Shults J., Cielo C.M., Kelly A.B., Elden L.M., Spergel J.M., Bradford R.M., Cornaglia M.A., Sterni L.M., Radcliffe J. (2022). A Trial of Intranasal Corticosteroids to Treat Childhood OSA Syndrome. Chest.

[34] Cielo C.M., Gungor A. (2016). Treatment Options for Pediatric Obstructive Sleep Apnea. Curr. Probl. Pediatr. Adolesc. Health Care.

[35] Kheirandish-Gozal L., Bandla H.P.R., Gozal D. (2016). Montelukast for Children with Obstructive Sleep Apnea: Results of a Double-blind Randomized Placebo-controlled Trial. Ann. Am. Thorac. Soc..

[36] Liming B.J., Ryan M., Mack D., Ahmad I., Camacho M. (2018). Montelukast and Nasal Corticosteroids to Treat Pediatric Obstructive Sleep Apnea: A Systematic Review and Meta-analysis. Otolaryngol. Neck Surg..

[37] Aschenbrenner D.S. (2020). New Boxed Warning for Singulair. AJN Am. J. Nurs..

[38] Evangelisti M., Barreto M., Di Nardo G., Del Pozzo M., Parisi P., Villa M.P. (2021). Systemic corticosteroids could be used as bridge treatment in children with obstructive sleep apnea syndrome waiting for surgery. Sleep Breath..

[39] Don D.M., Goldstein N.A., Crockett D.M., Ward S.D. (2005). Antimicrobial Therapy for Children With Adenotonsillar Hypertrophy and Obstructive Sleep Apnea: A Prospective Randomized Trial Comparing Azithromycin vs Placebo. Otolaryngol. Neck Surg..

[40] Zuliani G., Carron M., Gurrola J., Coleman C., Haupert M., Berk R., Coticchia J. (2006). Identification of adenoid biofilms in chronic rhinosinusitis. Int. J. Pediatr. Otorhinolaryngol..

[41] Nath A., Emani J., Suskind D.L., Baroody F.M. (2013). Predictors of Persistent Sleep Apnea After Surgery in Children Younger Than 3 Years. JAMA Otolaryngol. Neck Surg..

[42] Kearney T.C., Vazifedan T., Baldassari C.M. (2022). Adenotonsillectomy outcomes in obese adolescents with obstructive sleep apnea. J. Clin. Sleep Med..

[43] Lee S.Y., Guilleminault C., Chiu H.Y., Sullivan S.S. (2015). Mouth breathing, “nasal disuse”, and pediatric sleep-disordered breathing. Sleep Breath..

[44] Huang Y.-S., Guilleminault C. (2013). Pediatric Obstructive Sleep Apnea and the Critical Role of Oral-Facial Growth: Evidences. Front. Neurol..

[45] Guilleminault C., Huang Y.S. (2018). From oral facial dysfunction to dysmorphism and the onset of pediatric OSA. Sleep Med. Rev..

[46] Amin R., Al-Saleh S., Narang I. (2015). Domiciliary noninvasive positive airway pressure therapy in children. Pediatr. Pulmonol..

[47] Yui M.S., Tominaga Q., Lopes B.C.P., Eckeli A.L., Rabelo F.A.W., Küpper D.S., Valera F.C.P. (2019). Nasal vs. oronasal mask during PAP treatment: A comparative DISE study. Sleep Breath..

[48] Castro-Codesal M.L., Olmstead D.L., MacLean J.E. (2019). Mask interfaces for home non-invasive ventilation in infants and children. Paediatr. Respir. Rev..

[49] Ramirez A., Khirani S., Aloui S., Delord V., Borel J.-C., Pépin J.-L., Fauroux B. (2013). Continuous positive airway pressure and noninvasive ventilation adherence in children. Sleep Med..

[50] Atag E., Krivec U., Ersu R. (2020). Non-invasive Ventilation for Children With Chronic Lung Disease. Front. Pediatr..

[51] Xanthopoulos M.S., Williamson A.A., Tapia I.E. (2021). Positive airway pressure for the treatment of the childhood obstructive sleep apnea syndrome. Pediatr. Pulmonol..

[52] Parmar A., Baker A., Narang I. (2019). Positive airway pressure in pediatric obstructive sleep apnea. Paediatr. Respir. Rev..

[53] Harsch I.A., Pour Schahin S., Radespiel-Tröger M., Weintz O., Jahreiß H., Fuchs F.S., Wiest G.H., Hahn E.G., Lohmann T., Konturek P.C. (2004). Continuous Positive Airway Pressure Treatment Rapidly Improves Insulin Sensitivity in Patients with Obstructive Sleep Apnea Syndrome. Am. J. Respir. Crit. Care Med..

[54] Katz S.L., MacLean J., Hoey L., Horwood L., Barrowman N., Foster B., Hadjiyannakis S., Legault L., Bendiak G.N., Kirk V.G. (2017). Insulin Resistance and Hypertension in Obese Youth With Sleep-Disordered Breathing Treated With Positive Airway Pressure: A Prospective Multicenter Study. J. Clin. Sleep Med..

[55] Johnstone S.J., Tardif H.P., Barry R.J., Sands T. (2001). Nasal bilevel positive airway pressure therapy in children with a sleep-related breathing disorder and attention-deficit hyperactivity disorder: Effects on electrophysiological measures of brain function. Sleep Med..

[56] Hady K.K., Okorie C.U.A. (2021). Positive Airway Pressure Therapy for Pediatric Obstructive Sleep Apnea. Children.

[57] Oyegbile-Chidi T. (2022). Continuous Positive Airway Pressure Use for Obstructive Sleep Apnea in Pediatric Patients. Sleep Med. Clin..

[58] Nixon G.M., Mihai R., Verginis N., Davey M.J. (2011). Patterns of Continuous Positive Airway Pressure Adherence during the First 3 Months of Treatment in Children. J. Pediatr..

[59] Bhattacharjee R., Benjafield A.V., Armitstead J., Cistulli P.A., Nunez C.M., Pepin J.-L.D., Woehrle H., Yan Y., Malhotra A. (2019). Adherence in children using positive airway pressure therapy: A big-data analysis. Lancet Digit. Health.

[60] Watach A.J., Xanthopoulos M.S., Afolabi-Brown O., Saconi B., Fox K.A., Qiu M., Sawyer A.M. (2020). Positive airway pressure adherence in pediatric obstructive sleep apnea: A systematic scoping review. Sleep Med. Rev..

[61] Mihai R., Vandeleur M., Pecoraro S., Davey M.J., Nixon G.M. (2017). Autotitrating CPAP as a Tool for CPAP Initiation for Children. J. Clin. Sleep Med..

[62] Cielo C.M., Hernandez P., Ciampaglia A.M., Xanthopoulos M.S., Beck S.E., Tapia I.E. (2020). Positive Airway Pressure for the Treatment of OSA in Infants. Chest.

[63] Kang E.K., Xanthopoulos M.S., Kim J.Y., Arevalo C., Shults J., Beck S.E., Marcus C.L., Tapia I.E. (2019). Adherence to Positive Airway Pressure for the Treatment of Obstructive Sleep Apnea in Children With Developmental Disabilities. J. Clin. Sleep Med..

[64] Marcus C.L., Rosen G., Ward S.L.D., Halbower A.C., Sterni L., Lutz J., Stading P.J., Bolduc D., Gordon N. (2006). Adherence to and Effectiveness of Positive Airway Pressure Therapy in Children With Obstructive Sleep Apnea. Pediatrics.

[65] Bakker J.P., Weaver T.E., Parthasarathy S., Aloia M.S. (2019). Adherence to CPAP: What should we be aiming for, and how can we get there?. Chest.

[66] Sawunyavisuth B., Ngamjarus C., Sawanyawisuth K. (2021). Any Effective Intervention to Improve CPAP Adherence in Children with Obstructive Sleep Apnea: A Systematic Review. Glob. Pediatr. Health.

[67] Mulholland A., Mihai R., Ellis K., Davey M.J., Nixon G.M. (2021). Paediatric CPAP in the digital age. Sleep Med..

[68] Gozal D., Tan H.-L., Kheirandish-Gozal L. (2020). Treatment of Obstructive Sleep Apnea in Children: Handling the Unknown with Precision. J. Clin. Med..

[69] Shoemaker M.T., Pierce M.R., Yoder B.A., DiGeronimo R.J. (2007). High flow nasal cannula versus nasal CPAP for neonatal respiratory disease: A retrospective study. J. Perinatol..

[70] Dani C., Pratesi S., Migliori C., Bertini G. (2009). High flow nasal cannula therapy as respiratory support in the preterm infant. Pediatr. Pulmonol..

[71] Joseph L., Goldberg S., Shitrit M., Picard E. (2015). High-Flow Nasal Cannula Therapy for Obstructive Sleep Apnea in Children. J. Clin. Sleep Med..

[72] Nishimura M. (2016). High-Flow Nasal Cannula Oxygen Therapy in Adults: Physiological Benefits, Indication, Clinical Benefits, and Adverse Effects. Respir. Care.

[73] Narang I., Carberry J.C., Butler J.E., Gandevia S.C., Chiang A.K., Eckert D.J. (2021). Physiological responses and perceived comfort to high-flow nasal cannula therapy in awake adults: Effects of flow magnitude and temperature. J. Appl. Physiol..

[74] Kushida C.A., Halbower A.C., Kryger M.H., Pelayo R., Assalone V., Cardell C.-Y., Huston S., Willes L., Wimms A.J., Mendoza J. (2014). Evaluation of a New Pediatric Positive Airway Pressure Mask. J. Clin. Sleep Med..

[75] McGinley B.M., Patil S.P., Kirkness J.P., Smith P.L., Schwartz A.R., Schneider H. (2007). A Nasal Cannula Can Be Used to Treat Obstructive Sleep Apnea. Am. J. Respir. Crit. Care Med..

[76] McGinley B., Halbower A., Schwartz A.R., Smith P.L., Patil S.P., Schneider H. (2009). Effect of a High-Flow Open Nasal Cannula System on Obstructive Sleep Apnea in Children. Pediatrics.

[77] Fishman H., Al-Shamli N., Sunkonkit K., Maguire B., Selvadurai S., Baker A., Amin R., Propst E.J., Wolter N.E., Eckert D.J. (2023). Heated humidified high flow nasal cannula therapy in children with obstructive sleep apnea: A randomized cross-over trial. Sleep Med..

[78] Hawkins S., Huston S., Campbell K., Halbower A. (2017). High-Flow, Heated, Humidified Air Via Nasal Cannula Treats CPAP-Intolerant Children With Obstructive Sleep Apnea. J. Clin. Sleep Med..

[79] Ignatiuk D., Schaer B., McGinley B. (2020). High flow nasal cannula treatment for obstructive sleep apnea in infants and young children. Pediatr. Pulmonol..

[80] Verhelst E., Clinck I., Deboutte I., Vanderveken O., Verhulst S., Boudewyns A. (2019). Positional obstructive sleep apnea in children: Prevalence and risk factors. Sleep Breath..

[81] Selvadurai S., Voutsas G., Massicotte C., Kassner A., Katz S.L., Propst E.J., Narang I. (2020). Positional obstructive sleep apnea in an obese pediatric population. J. Clin. Sleep Med..

[82] Menon A., Kumar M. (2013). Influence of body position on severity of obstructive sleep apnea: A systematic review. ISRN Otolaryngol..

[83] Saigusa H., Suzuki M., Higurashi N., Kodera K. (2009). Three-dimensional Morphological Analyses of Positional Dependence in Patients with Obstructive Sleep Apnea Syndrome. Anesthesiology.

[84] Squier S.B., Patil S.P., Schneider H., Kirkness J.P., Smith P.L., Schwartz A.R., Lambeth C., Kolevski B., Kairaitis K., Amatoury J. (2010). Effect of end-expiratory lung volume on upper airway collapsibility in sleeping men and women. J. Appl. Physiol..

[85] Takahashi S., Ono T., Ishiwata Y., Kuroda T. (1999). Effect of changes in the breathing mode and body position on tongue pressure with respiratory-related oscillations. Am. J. Orthod. Dentofac. Orthop..

[86] Xiao L., Baker A., Voutsas G., Massicotte C., Wolter N.E., Propst E.J., Narang I. (2021). Positional device therapy for the treatment of positional obstructive sleep apnea in children: A pilot study. Sleep Med..

[87] Guimarães K.C., Drager L.F., Genta P.R., Marcondes B.F., Lorenzi-Filho G. (2009). Effects of Oropharyngeal Exercises on Patients with Moderate Obstructive Sleep Apnea Syndrome. Am. J. Respir. Crit. Care Med..

[88] Villa M.P., Evangelisti M., Martella S., Barreto M., Del Pozzo M. (2017). Can myofunctional therapy increase tongue tone and reduce symptoms in children with sleep-disordered breathing?. Sleep Breath..

[89] Sutherland K., Weichard A.J., Davey M.J., Horne R.S., Cistulli P.A., Nixon G.M. (2019). Craniofacial photography and association with sleep-disordered breathing severity in children. Sleep Breath..

[90] Fagundes N.C.F., Gianoni-Capenakas S., Heo G., Flores-Mir C. (2022). Craniofacial features in children with obstructive sleep apnea: A systematic review and meta-analysis. J. Clin. Sleep Med..

[91] Camacho M., Chang E.T., Song S.A., Abdullatif J., Zaghi S., Pirelli P., Certal V., Guilleminault C. (2017). Rapid maxillary expansion for pediatric obstructive sleep apnea: A systematic review and meta-analysis. Laryngoscope.

[92] Hoxha S., Kaya-Sezginer E., Bakar-Ates F., Köktürk O., Toygar-Memikoğlu U. (2018). Effect of semi-rapid maxillary expansion in children with obstructive sleep apnea syndrome: 5-month follow-up study. Sleep Breath..

[93] Fernández-Barriales M., de Mendoza I.L.-I., Pacheco J.J.A.-F., Aguirre-Urizar J.M. (2022). Rapid maxillary expansion versus watchful waiting in pediatric OSA: A systematic review. Sleep Med. Rev..

[94] Jacobs S., Mylemans E., Ysebaert M., Vermeiren E., De Guchtenaere A., Heuten H., Bruyndonckx L., De Winter B.Y., Van Hoorenbeeck K., Verhulst S.L. (2021). The impact of obstructive sleep apnea on endothelial function during weight loss in an obese pediatric population. Sleep Med..

[95] Verhulst S.L., Van Gaal L., De Backer W., Desager K. (2008). The prevalence, anatomical correlates and treatment of sleep-disordered breathing in obese children and adolescents. Sleep Med. Rev..

[96] Van de Perck E., Van Hoorenbeeck K., Verhulst S., Saldien V., Vanderveken O., Boudewyns A. (2020). Effect of body weight on upper airway findings and treatment outcome in children with obstructive sleep apnea. Sleep Med..

[97] Van Eyck A., De Guchtenaere A., Van Gaal L., De Backer W., Verhulst S.L., Van Hoorenbeeck K. (2018). Clinical Predictors of Residual Sleep Apnea after Weight Loss Therapy in Obese Adolescents. J. Pediatr..

[98] Roche J., Isacco L., Masurier J., Pereira B., Mougin F., Chaput J.-P., Thivel D. (2020). Are obstructive sleep apnea and sleep improved in response to multidisciplinary weight loss interventions in youth with obesity? A systematic review and meta-analysis. Int. J. Obes..

[99] Kelly A.S., Auerbach P., Barrientos-Perez M., Gies I., Hale P.M., Marcus C., Mastrandrea L.D., Prabhu N., Arslanian S. (2020). A randomized, controlled trial of liraglutide for adolescents with obesity. N. Engl. J. Med..

[100] Dhillon S. (2022). Phentermine/Topiramate: Pediatric First Approval. Pediatr. Drugs.

[101] Furbetta N., Gragnani F., Cervelli R., Guidi F., Furbetta F. (2020). Teenagers with obesity: Long-term results of laparoscopic adjustable gastric banding. J. Pediatr. Surg..

[102] Mashaqi S., Patel S.I., Combs D., Estep L., Helmick S., Machamer J., Parthasarathy S. (2021). The Hypoglossal Nerve Stimulation as a Novel Therapy for Treating Obstructive Sleep Apnea—A Literature Review. Int. J. Environ. Res. Public Health.

[103] Skotko B.G., Macklin E.A., Muselli M., Voelz L., McDonough M.E., Davidson E., Allareddy V., Jayaratne Y.S.N., Bruun R., Ching N. (2017). A predictive model for obstructive sleep apnea and Down syndrome. Am. J. Med. Genet. Part A.

[104] Liu P., Kong W., Fang C., Zhu K., Dai X., Meng X. (2022). Hypoglossal nerve stimulation in adolescents with down syndrome and obstructive sleep apnea: A systematic review and meta-analysis. Front. Neurol..

[105] Cielo C.M., Tapia I.E. (2023). What’s New in Pediatric Obstructive Sleep Apnea?. Sleep Med. Clin..

[106] Stenerson M.E., Yu P.K., Kinane T.B., Skotko B.G., Hartnick C.J. (2021). Long-term stability of hypoglossal nerve stimulation for the treatment of obstructive sleep apnea in children with Down syndrome. Int. J. Pediatr. Otorhinolaryngol..

[107] Taranto-Montemurro L., Edwards B.A., Sands S.A., Marques M., Eckert D.J., White D.P., Wellman A. (2016). Desipramine Increases Genioglossus Activity and Reduces Upper Airway Collapsibility during Non-REM Sleep in Healthy Subjects. Am. J. Respir. Crit. Care Med..

[108] Liu X., Sood S., Liu H., Horner R.L. (2005). Opposing muscarinic and nicotinic modulation of hypoglossal motor output to genioglossus muscle in rats in vivo. J. Physiol..

